# Functional and Phenotypic Plasticity of CD4^+^ T Cell Subsets

**DOI:** 10.1155/2015/521957

**Published:** 2015-10-25

**Authors:** Tiffany Caza, Steve Landas

**Affiliations:** Department of Pathology, Upstate Medical University, 750 East Adams Street, Syracuse, NY 13210, USA

## Abstract

The remarkable plasticity of CD4^+^ T cells allows individuals to respond to environmental stimuli in a context-dependent manner. A balance of CD4^+^ T cell subsets is critical to mount responses against pathogen challenges to prevent inappropriate activation, to maintain tolerance, and to participate in antitumor immune responses. Specification of subsets is a process beginning in intrathymic development and continuing within the circulation. It is highly flexible to adapt to differences in nutrient availability and the tissue microenvironment. CD4^+^ T cell subsets have significant cross talk, with the ability to “dedifferentiate” given appropriate environmental signals. This ability is dependent on the metabolic status of the cell, with mTOR acting as the rheostat. Autoimmune and antitumor immune responses are regulated by the balance between regulatory T cells and Th_17_ cells. When a homeostatic balance of subsets is not maintained, immunopathology can result. CD4^+^ T cells carry complex roles within tumor microenvironments, with context-dependent immune responses influenced by oncogenic drivers and the presence of inflammation. Here, we examine the signals involved in CD4^+^ T cell specification towards each subset, interconnectedness of cytokine networks, impact of mTOR signaling, and cellular metabolism in lineage specification and provide a supplement describing techniques to study these processes.

## 1. An Introduction to CD4^**+**^ T Cell Diversity

Production of a diverse repertoire of antigen-specific CD4^+^ T lymphocytes is essential for a host to respond to emerging microbial threats to create memory for heightened secondary responses to previously encountered pathogens and to suppress immune responses after microbial clearance to avoid tissue damage resulting from excessive or protracted inflammation [1]. Plasticity of CD4^+^ T cells is required to maintain immunocompetence after the thymic involution in adulthood [2]. Varying functional CD4^+^ T cell clones are also required to operate immune responses in different tissues as well as to produce high-affinity, class-switched immunoglobulin [3].

It is hypothesized that CD4^+^ T cells undergo subset specification but not lineage determination [3]. CD4^+^ T cells mature to form subsets with specified phenotypes and differences in cytokine production but fall short of terminal differentiation. Specification is a reversible maturation process that allows CD4^+^ T cells to undergo alternate fates, depending on environmental signals received. Signals contributing to subset specification include the prevailing cytokine environment, cytokine receptor expression profiles, transcription factor expression, and differential chromatin remodeling of loci that regulate production of effector cytokines [4].

Naïve CD4^+^ T cells undergo specification by many innate immune signals, including cytokines, chemokines, and inflammasome activation, which result in activation of signal transducers and activators of transcription, subsequent activation of lineage-specific transcription factors, cytokine production, and epigenetic adjustments at the cytokine loci to result in commitment to a given lineage.

Once a naïve T cell is primed by signals received from an antigen-presenting cell, proliferation occurs before lineage specification begins. If differentiation of CD4^+^ T cells occurred early after priming, peripheral CD4^+^ T cells would be restricted with binary options, being able to turn on or repress production of only a restricted subset of cytokines [5]. Subset determination occurring after clonal proliferation is consistent with an activated CD4^+^ naïve T cell producing many diverse progeny with pleiotropic, distinct fates, producing a highly flexible, dynamic, and context-driven CD4^+^ T cell repertoire [5].

Surprisingly, CD4^+^ T cell that has undergone lineage specification is capable of adopting alternate fates when innate immune signals change. The molecular basis for cytokine memory involves imprinting gene loci encoding cytokines by demethylation of DNA or histone acetylation as cells progress through S phase, so stable patterns of gene expression occur with an increasing number of cell divisions [6]. Yet, later chromatin remodeling occurs within CD4^+^ T cells to turn on new cytokine production profiles [5].

In this review, we will first examine functional differences between CD4^+^ T cell subsets and their lineage specification. A focus on the interconnectedness among pathways of maturation will follow with a presentation of experimental evidence supporting the hypothesis that CD4^+^ T cells maintain plasticity. The role of mTOR and cellular metabolism in T cell differentiation and function will be discussed. Finally, the impact of CD4^+^ T cell subsets in immunopathology and in antitumor immune responses will be considered.

## 2. T Cell Subsets and Lineage Specification

### 2.1. CD4^+^ T Cell Diversity Begins during Development

Diversity of the CD4^+^ T cell repertoire begins during intrathymic development. Thymocyte differentiation produces a diversity of CD4^+^ T cells with varying antigen specificities through *β*-selection, followed by *α*-chain rearrangement to form diverse *αβ* TCR specificities [27]. CD4 lineage selection is mediated through interaction of the T cell receptor (TCR) with class II MHC ligands. CD4^+^ T cell development is promoted by high TCR signal strength and signaling downstream of the TCR contributes to CD4 lineage commitment through association of Lck with the CD4 coreceptor and MAP kinase signaling to favor maintenance of CD4 expression with concurrent downregulation of CD8 [28]. CD4 commitment is mediated through induction of the transcription factor, T helper-inducing POZ/Kruppel-like factor (Th-POK), by GATA3 which represses Runx3 to release activity of the CD4 silencer [10, 29].

The lineage decision of commitment to the CD4^+^ or CD8^+^ T cell lineage was thought to be committed and inflexible, although it is now understood that there is a high degree of latitude in the CD4^+^ T cell compartment [3]. Lineage commitment is regulated not only through positive and negative selection but also through additional mechanisms. Helper-deficient (HD) mice, which lack the ability to produce CD4^+^ T cells, have spontaneous redirection of MHC class II-restricted T cells to the CD8^+^ lineage. The factor identified to be responsible for the redirection of the MHCII-restricted T cells to the CD8^+^ lineage was a mutation within the transcription factor, ThPOK. Wild-type ThPOK suppresses the cytolytic gene expression profiles in CD4^+^ T cells to induce lineage maturation and is both required and sufficient for maturation of the CD4^+^ T cell lineage. It was recently identified that antigen-experienced CD4^+^ T cells can turn off ThPOK to reactivate genes of the CD8 lineage, showing that this maturation step in intrathymic development is not fixed [30].

Early after priming by the innate immune response, CD4^+^ T cells are able to undergo conversion to an alternate subset through cytokine and chemokine receptor signaling, which induces changes in transcription factor expression [3]. T cell subset specification is influenced by interactions with dendritic cells (DCs) or peritoneal macrophages, the dose and form of presented antigen, the affinity of peptide-TCR interaction, cytokines, and costimulatory interactions [4, 31]. Asymmetric cell division during the DP to SP transition in intrathymic development also influences CD4^+^ T cell lineage decisions as daughter cells may “inherit” unequal shares of signaling molecules due to altered positioning across autocrine or paracrine chemokine gradients, influenced in part by Notch signaling [10]. Notch binding DLL 1 and 4 ligands promote lineage commitment to the Th_1_ subset, while Notch-Jagged interactions result in Th_2_ specification [7].

During at least the first several rounds of cell division under polarizing conditions, Th subset populations are heterogeneous, have low frequencies of cytokine producing cells, and have reversible phenotypes and effector cytokine production [4]. CD4^+^ T cells are plastic at this stage and beyond and are capable of switching their phenotypes to produce cytokines based on their activation status, environment, and metabolism. Reversibility is possible because the lineage-specific transcription factors that act as master regulators for subset specification are not fully repressed in other lineages but carry both permissive and repressive epigenetic marks or bivalency [3]. Bivalent epigenetic marks allow for rapid transition between active transcription and repressed transcription [32]. CD4^+^ T cells maintain flexibility in expression of genes encoding transcription factors that regulate cytokine loci, allowing adaptation to altered programs of cytokine expression in a potentially damaging inflammatory milieu [12].

### 2.2. CD4^+^ T Cell Subsets

Identification and characterization of CD4^+^ T cell lineage subsets began nearly three decades ago with the landmark papers of Mosmann et al., which described and identified two CD4^+^ T cell subsets, Th_1_ and Th_2_ [8, 33–35]. Subsets of CD4^+^ T cells were identified based on production of cytokines in specific responses to antigen or generalized stimulation with Con A [33, 34]. It was identified that Th_1_ cells produce IL-2 and IFN*γ*, while Th_2_ cells produced IL-4, IL-5, preproenkephalin, and p600 [34]. Both clones produced IL-3, GM-CSF, and TNF. They further defined the role of Th_1_ cells in mediating antigen-specific, MHC restricted, delayed type hypersensitivity reactions for a variety of antigens, while this ability was absent in Th_2_ cells [8]. Additionally, it was shown that Th_2_ cells produce a “cytokine synthesis inhibitory factor” capable of inhibiting Th_1_ cytokine production without a change in viability of the Th_1_ clones [35]. These discoveries first identified that CD4^+^ T cells were functionally and phenotypically heterogeneous and capable of cross talk.

One mechanism in which CD4^+^ T cells undergo subset specialization is through responding to cytokine signals produced in the innate immune response, inducing activation of lineage-specific transcription factors that result in production of a set of effector cytokines [4]. The initial priming cytokines are those produced by antigen presenting cells (APCs) [5]. Activated APCs deliver three types of signals required for the clonal expansion and maturation of CD4^+^ T cells [31]. The first signal is mediated by the peptide-MHC interaction with the TCR. The second involves costimulatory interactions between the APC and the T cell. The third signal directs differentiation of naïve T cells to effector T cell subsets through cytokines, Ras-MAPK signaling, and Notch ligand interactions [31, 36]. Pathogen recognition by macrophages and dendritic cells of the innate immune response initiates a signaling program that stimulates T lymphocytes and initiates adaptive responses [37]. The fate of a naïve T cell to undergo subset differentiation depends upon cytokine signaling and activation of proteins of the signal transducer and activation of transcription family (STATs). STAT activation is mediated by Janus kinases (JAKs) that are induced during the initial priming period. JAK-STAT triggering leads to activation of lineage-specific transcription factors, which results in expression of effector cytokines [4].

The differential function of CD4^+^ T cells is determined through which specific cytokines they produce [38]. Cytokines responsible for induction of CD4^+^ T cell differentiation, lineage specific transcription factors activated during subset specification, effector cytokines produced, and general functions of T cell subsets are summarized in Table 1.

Known CD4^+^ T cell subsets include Th_1_, Th_2_, Th_17_, Th_9_, Th_25_, T follicular helper cells (T_FH_), and regulatory T cells (T_reg_). Th_1_ cells are produced in response to intracellular pathogens (including parasites, viruses, and intracellular bacteria) and mediate cell-mediated immunity and delayed-type hypersensitivity reactions [8]. The Th_1_ program is induced by IFN*γ* produced by natural killer (NK) and dendritic cells, which activates STAT1, resulting in activation of the lineage-specific transcription factor T-bet [9]. IL-27, a cytokine of the IL-12 family, also contributes to STAT1 phosphorylation and T-bet activation. T-bet expression increases production of the IL-12 receptor, which activates STAT4, leading to activation of IFN*γ* transcription and subsequent IFN*γ* production [7]. This serves as positive feedback, stimulating more naïve T cell clones to undergo Th_1_ specification to polarize the immune response towards fighting an intracellular pathogen. Th_1_ cells also produce TNF*α* and lymphotoxin, cytokines which trigger neutrophil chemotaxis and macrophage activation to potentiate innate immune reactions [38, 39]. Th_1_ cells also help B cells in antibody class-switching to produce high-affinity IgG for opsonization of an offending pathogen [40].

Th_2_ specification is required for B cell help in humoral immunity and elimination of extracellular microbes and intestinal helminthes [5, 40]. Th_2_ cells are involved in antibody class-switching to produce IgE which can provoke or sustain allergic reactions [8, 36, 39]. Differentiation of the Th_2_ subset requires IL-4 produced by Notch ligand activation of dendritic cells which, in turn, induces STAT6, which activates the lineage-specifying transcription factor, GATA-3 [36]. GATA-3 activates transcription of the Th_2_ cytokine cluster leading to IL-4, IL-5, and IL-13 production. Th_2_ cytokines provide positive feedback for maturation of naïve T cells to the Th_2_ lineage and inhibit Th_1_ development [39] by the homeostatic cytokine IL-10. Th_2_ cells also heighten the innate immune response through activation of macrophages by induction of IL-4 and macrophage activating factor (MAF) [5].

Th_17_ cells provide protection against bacteria and fungi at mucosal surfaces and confer coverage of some microbes that are not targeted in Th_1_ or Th_2_ responses, including, but not limited to,* Mycobacterium tuberculosis*,* Bacteroides fragilis*, and* Klebsiella pneumoniae* [15]. Induction of the Th_17_ lineage occurs when IL-6, IL-23, and TGF*β* are present in the inflammatory milieu without IL-4 or IL-12 (which promote Th_2_ or Th_1_ responses, resp.) [41, 42]. Toll-like receptor signaling, leading to MyD88 signaling, is another innate immune signal fostering Th_17_ differentiation [43]. IL-6 promotes STAT3, which induces retinoic orphan receptor (ROR) transcription factors, ROR*α* and ROR*γ*T, leading to production of Th_17_ cytokines IL-17, IL-17F, and IL-22 [44, 45].

Mucosal immunity is provided through Th_9_, Th_22_, and IL-25 producing cells [46]. Th_9_ cells provide protection against intestinal helminth infections [13]. IL-9 producing cells are proinflammatory as they stimulate proliferation and inhibit apoptosis of hematopoietic cells and also activate Th_17_ cells [47]. This is due to stimulation of Jak1 by IL-9, resulting in activation of STATs 1, 3, and 5. Th_9_ cells undergo a maturation program similar to Th_2_ cells, with IL-4 inducing STAT6 activation and produce the Th_2_ cytokines IL-9 and IL-10, but, unlike Th_2_ cells, they require TGF*β* for maturation [13, 48]. The lineage-specific transcription factor for Th_9_ development may be the activator protein 1 family transcription factor, BATF, leading to a transcriptional program which results in increased IL-9 and IL-10 production [13, 49]. Th_22_ cells are CD4^+^ T cells that are phenotypically and functionally related to Th_17_ cells that participate in wound repair and in protection against bacterial, viral, and fungal infections at epithelial surfaces, including the skin and GI tract [21]. They prevent translocation of microbes across epithelia, which limit the extent of infection [18]. Th_22_ specification is promoted by IL-6 and TNF-*α*, which induces STAT3, and expression of the aryl hydrocarbon receptor [19]. This parallels Th_17_ maturation, and numerous phenotypic markers are expressed in common between Th_17_ and Th_22_ cells, including CCR6, CCR4, dipeptidyl peptidase IV, CD26, and CD90 [20]. CCR10 is also expressed on Th_22_ cells, distinct from Th_17_ [20]. Th_22_ cells produce IL-22, IL-13, fibroblast growth factor, CCL15, CCL17, and TNF*α* at epithelial surfaces. IL-22, an IL-10 family cytokine, production is not unique to Th22 cells but is also produced by Th_1_ and Th_17_ cells; however, Th_22_ cells can produce IL-22 in the absence of IFN*γ* or IL-17 [20]. IL-25-producing cells may represent a new subset, Th_25_ cells, which stimulate nonlymphoid cells to produce effector cytokines in response to extracellular pathogens [22]. They are induced by the transcription factor Act1, but can be derived from the Th_2_ lineage [12, 46]. IL-25-producing cells and the Th_2_ subset may be linked as IL-4 is required for production of both cell types and IL-25 enhances production of Th_2_ cytokines, inducing IL-4, IL-5, and IL-13 secretion [12, 50].

T follicular helper cells (T_FH_) improve B cell class-switching for immunoglobulin production and guide B cells into germinal centers by chemotaxis mediated by CXCR5 signaling [23]. T_FH_ cells require a strong TCR signal for induction, which is also required for T_reg_ responses [24, 51]. T_FH_ specification requires activation of the inducible costimulator (ICOS), a CD28-related costimulatory signal provided by activated dendritic cells or B cells, which initiates transcription of the transcription factor MAF that transactivates IL-21 [24]. OX-40/CD134 ligation is another required costimulatory signal, which downregulates CTLA-4, a dominant suppressor of T cell activation [24]. IL-6 and STAT3 are required for T_FH_ development similar to Th_17_ cells, yet T_FH_ cells can be generated in the absence of Th_17_ cytokines, IL-17, IL-17F, or TGF*β* [23].

Suppression of immune responses and maintenance of peripheral tolerance is provided by T_reg_s [25]. T_reg_s are a heterogeneous population which includes thymic-derived natural T_reg_s (*n*T_reg_s), adaptive regulatory T cells involved in maintaining oral tolerance (Th_3_ cells), and T regulatory type 1 cells (Tr1 cells), induced by IFN*α* secreted by neighboring plasmacytoid dendritic cells (pDCs).* n*T_reg_s require a strong TCR signal, which is potentially self-reactive, for development [52]. They are generated with minimal costimulation, for T cell recognition of antigen without a strong second signal from a CD28 family member can provide induction of tolerance [52]. Differentiation of induced T_reg_s, Th_3_ cells, and Tr1 cells occurs in the periphery and requires high concentrations of TGF*β*, with the absence of proinflammatory cytokines [15]. Cell-cell contact and IL-10 secretion is required for suppressor function, mediated through STAT5-induced activation of the lineage-specific transcription factor Foxp3, with concurrent downregulation of the Th_17_ transcription factor ROR*γ*T [16]. Suppressor function of T_reg_s requires Foxp3 expression [53]. Reduced T_reg_ numbers and effector function occur in autoimmune diseases and complete deficiency of this subset results in a severe autoimmune disease, immune dysregulation, polyendocrinopathy, and enteropathy with X-linked inheritance (IPEX) syndrome [52].

A population of non-T_reg_ Foxp3-expressing CD4^+^ T cells has been identified, which is known as the “exFoxp3” T cell [54]. exFoxp3 cells have transient Foxp3 expression in an activated state, and these cells can accumulate at sites of inflammation. These represent effector T cells that gain Foxp3 expression and not conversion to the T_reg_ lineage. A small population of T_reg_s with loss of Foxp3 expression while maintaining commitment to the T_reg_ lineage also exists. This is through demethylation at the TSDR locus, which retains memory of its suppressor phenotype [54].

Central memory CD4^+^ T cells, created through initial priming and restimulation, consist of a heterogeneous population that is not lineage-committed, for memory responses are subject to manipulation under cytokine-polarizing conditions to adapt to new antigenic stimuli [55]. Effector memory CD4^+^ T cells are thought to have undergone lineage determination. Plasticity of the central memory population is essential for maintenance of specific CD4^+^ T cells after pathogen clearance, since 90–99 percent of Th_1_ or Th_2_ effector cells will undergo apoptosis after antigenic challenge [56].

## 3. Cross Talk and Flexibility in T Cell Subset Lineage Specification

Effector cytokines produced by CD4^+^ T cells provide positive feedback to increase further differentiation of naïve T cells to that lineage while inhibiting differentiation of opposing subsets [57]. Signal transduction pathways induced by cytokines and chemokines influence lineage commitment events through activation or repression of a subset-specific transcriptional program [58]. 

*Th*_*1*_ *and Th*_*2*_ *Subsets*. Commitment to the Th_1_ lineage inhibits Th_2_ development, and Th_2_ commitment inhibits Th_1_ responses [39]. IFN*γ* production by Th_1_ cells inhibits production of Th_2_ cytokines [39]. Likewise, IL-4 produced during Th_2_ specification inhibits production of IFN*γ* and IL-12, preventing differentiation of naïve CD4^+^ T cells to the Th_1_ lineage [39]. GATA3 expression by Th_2_ cells leads to upregulation of sphingosine kinase I expression and downregulation of STAT4, which inhibit Th_1_ development [59].

Plasticity occurs between the Th_1_ and Th_2_ lineages, and early after naïve CD4^+^ T cell activation, production of IFN*γ* and IL-4 can occur simultaneously [55]. Decreased expression of intracellular osteopontin by APCs with increased soluble osteopontin produced by T cells (increased soluble-to-intracellular osteopontin ratio) stimulates IL-12 production to promote Th_1_ lineage commitment [60]. With culture of CD4^+^ T cells in a Th_1_-promoting environment (containing IL-12 and anti-IL-4 antibody), the population of cells will polarize to produce IFN*γ*. Removal of the polarized CD4^+^ T cells into IL-4 containing medium promotes Th_2_ cytokine production, displaying the capacity of converting between the two phenotypes. In addition, forced overexpression of GATA3 in Th_1_ polarized cells results in conversion to a Th_2_ phenotype, while T-bet overexpression in Th_2_ polarized cells results in a Th_1_ phenotype [61]. Flexibility between Th_1_ and Th_2_ cytokine production is lost, however, with repeated stimulation and multiple rounds of cell division [6]. This is thought to be due to chromatin remodeling at cytokine loci to increase efficiency of effector cytokine production and inhibit opposing cytokine programs [62].

Intrachromosomal interactions through modifications of chromatin structure are also responsible for repression of the alternate lineage program [32]. Stimulation under Th_1_ or Th_2_ polarizing conditions results in altered chromatin accessibility after 4 to 6 cell divisions. In naïve T cells, the IFN*γ* locus is bivalent, poised for enhancing gene expression or transcriptional silencing, depending on which signals are received. Under a Th_2_ polarizing cytokine environment, permissive histone modifications are lost at the IFN*γ* locus by DNA methylation. Similarly, repressive methylation at the Th_2_ locus occurs during Th_1_ polarization [63]. In addition to intrachromosomal modifications, interchromosomal interactions exist between the IFN*γ* and Th_2_ cytokine clusters for negative regulation of the opposite lineage. Direct interaction between the IFN*γ* promoter and regulatory regions of the Th_2_ cytokine cluster cross-regulate one another [64]. Naïve T cells have the ability to express both Th_1_ and Th_2_ cytokines within hours of T cell activation due to the interaction of these two loci creating a chromatin hub configuration between the IFN*γ* promoter and the Th_2_ locus control region [64].

Expressions of Th_1_ and Th_2_ cytokines from a single cell, as well as environments rich in both Th_1_ and Th_2_ cytokines, further show flexibility in subset specification. When CD4^+^ T cells are stimulated* in vitro* with IL-12, they produce both IFN*γ* and IL-4 [65]. Yet, repeated stimulation will reduce the percentage of cells expressing this phenotype, suggesting that the double-positive cells represent a transition state of differentiation.

*In vivo* polarization experiments using model pathogens have also demonstrated interconversion between Th_1_ and Th_2_ cells. CD4^+^ T cells exposed to* Leishmania major* infection differentiate into the Th_1_ lineage and produce IFN*γ* while maintaining the capacity of interchanging into a Th_2_ phenotype when exposed to IL-2 and IL-4* ex vivo* [65]. There is the possibility, however, that reversal from a Th_1_ to Th_2_ phenotype may simply reflect the outgrowth of a population of uncommitted cells rather than dedifferentiation from a Th subset [65]. 

*T*_*reg*_ *and Th*_*17*_ *Subsets*. Th_17_ and T_reg_ subsets are in a homeostatic balance and are derived from a common precursor. CD4^+^ T cells with dual expression of Foxp3 and ROR*γ*T exist during early Th17 cell development and in naïve T cells after stimulation with TGF*β*. These Foxp3^+^ROR*γ*T^+^ expressing cells may occur as an intermediate during commitment to an effector lineage, T_reg_ or Th_17_ cells [66]. They mature into either a T_reg_ or Th_17_ cell depending on the cytokine profile in the environment [16]. IL-6, IL-21, IL-23, and low levels of TGF*β* support induction of ROR*γ*T and Th_17_ development. High levels of TGF*β*, retinoic acid, and IL-2 support T_reg_ commitment through activation of Foxp3 [15, 66]. Th_17_ cells can be converted from induced T_reg_ cell populations in the presence of the cytokines IL-6 and IL-1. When TGF*β* expression is high and IL-6 is present, a population of Foxp3^+^IL-17^+^ cells results. STAT3 phosphorylation in cells committing to a Th_17_ lineage inhibits TGF*β*-induced Foxp3 expression [32]. Additionally, ROR*γ*T directly interacts with exon 2 of the Foxp3 gene to suppress T_reg_ development and activates transcription of Th_17_ cytokines. Similarly, Foxp3 can bind ROR*γ*T to suppress IL-17 production [66]. Other coexpressed transcription factors influence the T_reg_ v. Th_17_ lineage branch point. These include interferon regulatory factor-4 and Runx1, which promote Th_17_ differentiation through interaction with the Foxp3 locus [29]. Both thymic-derived and peripherally induced T_reg_s express Helios [67, 68], which could be used to identify whether a Th_17_ cell was derived from a T_reg_ versus being induced from a naïve T cell precursor. Low levels of Helios expression can indicate T cell activation, while high expression suggests T_reg_ origin [69]. Proinflammatory cytokine production by Th_17_ cells inhibits generation of T_reg_ cells, and T_reg_ production of IL-10 suppresses Th_1_ and Th_17_ generation [14, 25]. T_reg_s antagonize Th_17_ function and reduce IL-17 production when it is no longer required for pathogen clearance to avoid tissue injury [16].

T_reg_ and Th_17_ cells are the predominant CD4^+^ T cells within tumor microenvironments [70]. T_reg_s suppress antitumor immune responses through promoting tolerance to the tumor by IL-10 production. In the presence of IL-6, TGF*β*, type I interferons, IL-12, and intact toll-like receptor signaling via MyD88, Th_17_ cell specification is induced from T_reg_ cells [70, 71]. Overall, the number of T_reg_s is increased in many cancers and has been shown in gastric adenocarcinoma [72], esophageal adenocarcinoma [72], squamous cell carcinoma (head and neck) [73–75], breast carcinoma [76], and non-small cell lung carcinoma [76]. Reduced numbers of intratumoral Th_17_ cells have been associated with poor prognosis in several tumor models [70]. Yet, in a recent lung adenocarcinoma model, K-ras^G12D^ expression promoted recruitment of Th_17_ cells to the tumor and increased tumor growth, with IL-17 blockade reducing tumor burden [77]. Whether an excess of T_reg_s or Th_17_ cells is pathogenic within a tumor could be context-dependent, based on the type of tumor, its oncogenic drivers, the microenvironment, and the immunocompetence (versus compromise) of the host.

Proinflammatory cytokine production by Th_17_ cells inhibits generation of T_reg_ cells, and T_reg_ production of IL-10 suppresses Th_1_ and Th_17_ generation [14, 25]. T_reg_s antagonize Th_17_ function and reduce IL-17 production when it is no longer required for pathogen clearance to avoid tissue injury [16]. The T_reg_ lineage may not be fixed, as T_reg_s have been identified to differentiate into Th_17_ or T_FH_ cells. Foxp3^+^ T cells in the thymus develop into Th_17_ cells and produce IL-17 when taken* ex vivo* and put into IL-6-containing medium. Additionally, over one-fourth of Th_17_ cells along the small intestine mucosa are thought to be derived from Foxp3^+^ iT_reg_s [15]. However, it is possible that differentiation into effector CD4^+^ T cell subsets could represent a population of activated T cells with aberrant Foxp3 expression (exFoxp3 cells) rather than T_reg_s themselves.

Whether plasticity of T_reg_s exists is under debate; however, there is known heterogeneity within T_reg_ populations. Three T_reg_ subsets have been identified, which have varying functions, defined by expression of CD45RA, CD25 levels, and Foxp3 [78]. Activated T_reg_s are CD45RA^−^CD25^hi^Foxp3^hi^ and show suppressor function; CD45RA^+^CD25^moderate^Foxp3^lo^ subset represents resting regulatory T cells without suppressor function; and CD45RA^−^CD25^moderate^Foxp3^lo^ cells are non-T_reg_ effector T cells that are capable of cytokine production, making IL-2, IL-17, or IFN*γ* [78]. In autoimmune conditions, including systemic lupus erythematosus and sarcoidosis, there is an increased ratio CD45RA^−^CD25^+^Foxp3^hi^ cells compared to CD45RA^−^CD25^+^Foxp3^lo^ cells, although the absolute number of T_reg_ cells is overall reduced. This ratio was found to be reversed in cancer [79].

*Th*_*17*_ *Compared to Th*_*1*_ *or Th*_*2*_. Th_17_ cells have bivalent expression of T-bet and GATA-3, allowing them to reprogram into either Th_1_ or Th_2_ cells [32, 80]. Th_17_ cells generated by TGF*β* and IL-6* in vitro* can convert into IL-12-producing Th_1_ or IL-4-producing Th_2_ cells when ongoing stimulation with proinflammatory cytokines is not sustained [1]. Th_1_ cells differentiate from Th_17_ cells* in vitro* when IL-12 is present in medium in the absence of IL-6. IL-17^+^IFN*γ* ^+^-producing cells may represent an intermediate state during Th_1_ development from Th_17_ precursors. T-bet and ROR*γ*T are coexpressed in this transition state, allowing maturation of either the Th_1_ or Th_17_ lineage [32].

A distinct population of Th_1_/Th_17_ cells has been identified, which are CD4^+^ T cells capable of producing IFN*γ*, GM-CSF, and IL-17 [81]. Th_1_/Th_17_ cells express both T-bet and RORC2 concurrently to allow for bivalent cytokine production. In an inflammatory environment, they can further become polarized to become Th_1_/exTh_17_ cells through loss of active transcription from the RORC2 locus. Th_1_/ex-Th_17_ cells produce IFN*γ* and GM-CSF but lost the ability to produce IL-17 [81]. Although T_reg_s are capable of transforming into IFN*γ* and IL-17 producing cells, Th_1_/Th_17_ cells are a separate entity, as expression of the transcription factor Helios (present in thymic-derived T_reg_s and to a lesser extent in peripherally induced T_reg_s) is absent or low in this subset.

Th_1_/Th_17_ cells have been associated with promoting autoimmune target organ damage in *β*-cells of human and animal models of type I diabetes mellitus [82] within synovial tissue of children with juvenile arthritis [83] and within the gastrointestinal tract of patients with Crohn's disease [84]. Blockade of IFN*γ* with monoclonal antibodies reduced pancreatic *β*-cell destruction in an animal model, supporting that Th_17_ cells that express a Th_1_-like phenotype are pathogenic [81]. 

*T*_*FH*_ *Compared to T*_*reg*_ *, Th*_*17*_ *, or Th*_*1*_. T_FH_ cells can be differentiated from T_reg_s, which requires loss of Foxp3 expression by T-B cell interaction with costimulation provided by CD40-CD40L interaction [24]. T_reg_s differentiate into T_FH_ cells in Peyer's patches to promote IgA production and mucosal immunity [1]. T_FH_ cells mature from TGF*β*-induced T_reg_ precursors in response to IL-21 and chemotaxis through CXCR5 signaling, which homes CD4^+^ T cells to follicles in secondary lymphoid tissue [23].

Th_17_ and T_FH_ cells both require STAT3 activation and costimulation mediated by ICOS, which increases activation of the transcription factor, c-MAF, involved in both Th_17_ and T_FH_ maturation [85]. In ICOS knockout mice, decreased c-MAF activation prevents development of T_FH_ cells and prevents defects in Th_17_ cells, which produce less IL-17 in response to stimulation [85]. IL-12 is required during early dendritic cell-mediated priming of both Th_1_ and T_FH_ cells, suggesting that T_FH_ and Th_1_ cells are derived under common innate immune signals [86]. 

*Th*_*2*_ *Compared to T*_*FH*_ *or Th*_*9*_. The same priming cytokine, IL-4, is required for the development of Th_2_, T_FH_, and Th_9_ subsets. Th_2_ cells can become T_FH_ cells through upregulation of CXCR5 expression for homing to germinal center follicles will continue to produce IL-4 but lose the ability to produce other Th_2_ cytokines, including IL-5 and IL-13 [13, 48]. T_FH_ cells do not have to progress through a Th_2_ stage in their development, as they still develop in GATA3 knockout mice [23].

Th_9_ cells can be derived from Th_2_ cells when TGF*β* is added to cells polarized with IL-4 in culture medium [13]. Continued activation of the Th_2_ cytokine, IL-4, or the Th_9_ cytokine, IL-9, in transgenic mice leads to the same phenotype, with asthma and bronchial hyperresponsiveness due to mucosal inflammation, also suggesting interconnectedness in their maturation [87].

**Figure 1:**
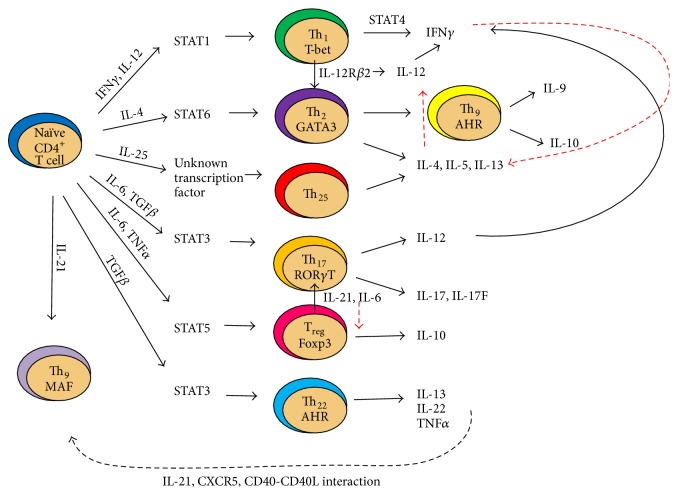
Cross talk between CD4^+^ T cell subsets mediated by effector cytokines.

A schematic diagram of interactions involved in CD4^+^ T cell lineage specification is provided in Figure 1.

Naïve CD4^+^ T cells undergo subset specification based on predominant cytokines and chemokines present within the environment. In the presence of IFN*γ* and IL-12, naïve CD4^+^ T cells upregulate STAT1 which induces specification to the Th_1_ lineage through T-bet expression [9, 58]. Th_1_ cells then produce IFN*γ* through STAT4 expression [88]. A positive feedback loop exists to further promote Th_1_ specification [89]. Conversely, Th_1_ cells inhibit Th_2_ specification through IFN*γ* production, as well as through T-bet expression. Th_2_ cells develop when IL-4 is present in the environment and require STAT6 upregulation to induce GATA-3 expression within the nucleus [90]. Th_2_-specified cells produce IL-4, IL-5, and IL-13. When TGF*β* is also present in the cytokine microenvironment, Th_9_ specification can occur [91]. As Th_1_ cells inhibit Th_2_ specification, Th_2_ cells also provide negative feedback to Th_1_ differentiation through suppressing IL-12 expression [89]. Th_25_ cells, induced by IL-25, represent a separate mechanism of GATA-3 induction and production of Th_2_-cytokines, IL-4, IL-5, and IL-13, which has been linked to reactive airway inflammation [46]. Th_25_ specification requires expression of the E3 ubiquitin ligase Act1 and the cognate receptor IL-17RB [46], related to the Th_17_ subset. The Th_17_ and T_reg_ lineages are interrelated, and both can be induced in the presence of TGF*β*, with Th_17_ specification preferred when IL-6 is also present. Th_17_ cells express lineage-specific transcription factor ROR*γ*T as well as other retinoic orphan receptors, which leads to production of Th_17_ cytokines, IL-12, IL-17, and IL-17F [92]. IL-12 produced by Th_17_ cells can provide feedback to promote Th_1_ specification [1]. In the presence of IL-6 or IL-21, T_reg_ cells can become Th_17_ cells [16]. IL-6 and TNF*α* can induce STAT3 activation and induce Th_22_ specification, a process that is dependent on the aryl hydrocarbon receptor [19]. In the presence of IL-21, CXCR5, and costimulation by CD40-CD40 ligand interaction, T_reg_s can undergo T follicular helper (T_FH_) specification [23].

### 3.1. Epigenetic Modifications of Cytokine Loci Determine Lineage Specification

Flexibility of CD4^+^ T cells to switch effector cytokine function in response to environmental signals depends on permissiveness of chromatin to transcription factor binding at loci encoding cytokines.

CD4^+^ T cells undergo multiple cell divisions before producing subset-specific effector cytokines, since maturation of CD4^+^ T cells into subsets requires continued stimulation under cytokine polarizing conditions, resulting in stable patterns of gene expression due to chromatin remodeling of loci encoding cytokine genes [1].

Epigenetic influences bring alterations of cytokine production profiles through mechanisms including alterations of chromatin structure through histone acetylation or DNA demethylation and modifications of microRNA activity [1]. Histone acetylation or demethylation of DNA brings decondensation (“opening”) of chromatin (or conversion of heterochromatin to euchromatin), resulting in increased access for transcription factor binding [4]. Modifications that occur when signals within the extracellular environment change allow a CD4^+^ T cell to redirect its differentiation program to provide flexibility to shift cytokine production for the optimal clearance of offending pathogens [1].

Genome-wide chromatin immunoprecipitation studies (CHIP) have characterized histone modifications which accompany changes in gene expression within CD4^+^ T cells. Trimethylation of lysine 4 at histone 3 (H3K4me3) occurs at promoter and enhancer elements of actively expressed genes. Trimethylation of lysine 27 of histone 3 (H3K27me3) represses a locus, and this repression is reversed by histone demethylases [3]. Presence of both of these histone modifications (bivalency of histone 3, or coexpression of H3K4me3 and H3K27me3) allows a gene promoter to become activated or silenced, depending on the signal received [1]. In addition, chromatin looping can bring regulatory elements to proximity with promoters of target genes enabling regulation of gene expression, a process mediated by CCCTC-binding factor, an insulator protein that binds proximal elements of a gene to prevent it from interacting with surrounding chromatin [32]. Looping allows transcriptional regulatory elements to reposition in the nucleus during T cell maturation to promote or repress transcription [7]. Chromatin looping allows the Th_2_ cytokine locus control region (LCR) to form a complex with the promoters that induce IL-4, IL-5, and IL-13 cytokine expression for activation of the Th_2_ transcriptional program [64].

In addition to changes in chromatin structure, interchromosomal associations allow for regulation of effector cytokine expression. Chromosome conformation capture studies (3C technique) have identified an interchromosomal interaction between the IFN*γ* promoter of human chromosome 10 and the LCR of the Th_2_ cytokine locus on chromosome 11 [64]. A chromatin hub configuration between Th_1_ and Th_2_ cytokine loci primes a naïve CD4^+^ T cell to produce either Th_1_ or Th_2_ cytokines 1 hour after TCR activation. After this early wave of cytokine production activation of STAT proteins is required for maintenance of the signal [62].

The lineage-specific transcription factors that direct Th_1_, Th_2_, and Th_17_ commitment (Tbx21, GATA3, and ROR*γ*T, resp.) carry bivalent epigenetic marks, signifying the possibility of subset specification reversibility. However, Foxp3 expression in T_reg_ cells is univalent, suggesting subset differentiation is possible but not reversible [32, 93].

### 3.2. mTOR as a Regulator of CD4^+^ Differentiation

The serine-threonine kinase, mammalian target of rapamycin (mTOR), is a candidate gene as a master regulator of CD4^+^ T cell differentiation and metabolism [94], which are interconnected. mTOR activation in CD4^+^ T cells has diverse roles in regulation of cell growth and proliferation, mRNA turnover and transcription, translation, regulation of vesicular traffic, autophagy and amino acid recycling, cytoskeletal reorganization, and control of cell size [95]. It exerts its function through phosphorylation of its target substrates, such as p70 S6 kinase and 4E-BP1 (which regulate translation), and DAP1 (which inhibits induction of autophagy). mTOR is positively regulated by the GTPase Rheb and negatively regulated by the tuberous sclerosis complex (TSC) [96].

mTOR activation results in increased maturation of CD4^+^ T cells into effector cells, with reduction of Foxp3 expression and T_reg_ generation [97]. Absence of mTOR at the DP phase in T cell development abrogates the ability to produce Th_1_, Th_2_, or Th_17_ cells and results in a high proportion of thymocytes maturing as T_reg_s [26]. This maturation defect is associated with decreased activation of STAT 4, 6, and 3, resulting in failure to upregulate lineage-specific transcription factors T-bet, GATA3, and ROR*γ*T [26]. Consistently, treatment with rapamycin (an mTOR inhibitor) results in thymic involution, decreased egress of T cells, and blockade of the DN to DP transition of T cell development [94]. Conversely, in effector CD4^+^ T cells, activation of the sphingosine-1-phosphate (S1P_1_) signaling pathway results in increased mTOR-Akt activation, which inhibits intrathymic generation and suppressor function of T_reg_s [98, 99]. Therefore, mTOR overexpression renders effector CD4^+^ T cells resistant to T_reg_ suppressor activity [94].

During effector CD4^+^ T cell activation, the PI3K-Akt-mTOR pathway is activated and regulates the effector T cell/T_reg_ cell fate decision [99]. mTOR activation transmits signaling through the IL-2 receptor, and IL-2 binding to the IL-2 receptor prevents T cell anergy, required for maintenance of effector T cells [25]. T_reg_ cells do not rely on mTOR activation for IL-2 receptor signaling but instead use an alternate PIM2-dependent pathway for IL-2 signaling, as STAT5 increases IL-2 through activation of PIM2. The activity of mTORC1 in comparison to mTORC2 determines whether naïve T cells differentiate into the Th_1_/Th_17_ lineage versus Th_2_ cells [100]. In the absence of Rheb, a kinase required for the function of mTORC1, Th_1_ and Th_17_ cells are not produced, as mTORC1 is required for Th_1_/Th_17_ specification [100]. In the absence of rictor, a component of mTORC2, Th_1_, and Th_17_ cells is generated but not Th_2_ cells. Therefore, mTORC1 activity is required to generate Th_1_ cells, while mTORC2 activity is required for Th_2_ specification [100].

Lineage determination by mTOR is through induction of changes in cellular metabolism through activation of its substrates (hypoxia-inducible factor, sterol regulatory element binding proteins 1 and 2), through regulation of mitochondrial function, and through negative regulation of autophagy [101]. mTOR activation results in a stimulation of glycolysis, pentose phosphate shunt pathway activity (oxidative branch), and lipid biosynthesis with a concurrent reduction in fatty acid oxidation [101]. Since mTOR stimulates glycolytic metabolism, it promotes Th_1_, Th_2_, and Th_17_ specification, as these subsets have high metabolic requirements [102]. As such, Th_1_, Th_2_, and Th_17_ cells also show high expression of the Glut1 receptor to facilitate increased glucose transport [102]. As mTORC1 supports an anabolic state with lipid biosynthesis over its utilization/oxidation, this results in reduced AMPK activity [103]. AMPK activity is critical to T_reg_ metabolism, supporting effector over T_reg_ cell specification when expression is low [103]. mTOR additionally impacts aerobic metabolism through control over mitochondrial function through regulation of mitochondrial number [104], transmembrane potential [105], oxygen consumption [105, 106], and autophagy [107–109].

A model of CD4^+^ T cell specification has been proposed based on differential activation of mTOR. In this model, the generation of effector CD4^+^ T cells from naïve CD4^+^ T cell precursors is dependent on mTOR-mediated induction of metabolic programs within CD4^+^ T cells [110]. mTOR^hi^ and mTOR^lo^ naïve CD4^+^ T cells were found to have different fates [111]. mTOR^hi^ CD4^+^ T cells will become effector cells, while mTOR^lo^ CD4^+^ T cells represent a long-lived CD4^+^ T cell population, with expression of Bcl-2, CD62L, and CD25 and a higher propensity to develop into T_reg_s [111]. Interestingly, the mTOR^lo^ and mTOR^hi^ naïve T cell populations can be separated based on their size, with the mTOR^hi^ population having increased cell size [111].

Although mTOR activity is critical to the regulation of CD4^+^ T cell development, specification, and metabolism, its hyperactivation is pathogenic. mTOR complex I is overexpressed in autoimmune diseases, genetic cancer syndromes, and obesity which correlates with a reduction in suppressor T_reg_s [112]. mTOR stimulates aerobic glycolysis, which promotes the Warburg effect within tumors [112]. mTORC1 activity increases Th_17_ cell number and reduces T_reg_s in systemic lupus erythematosus (SLE) [113, 114]. Overexpression of mTOR results in defects in macroautophagy [105, 115], which is pathogenic through mitochondrial dysfunction, ATP depletion, and increased oxidative stress [116]. mTOR inhibitors, such as rapamycin, are therefore therapeutic through inhibiting aberrant mTOR activity in the treatment of autoimmunity and malignancies [117–119].

## 4. Conclusion

As mentioned, naïve CD4^+^ T cells mature into Th_1_, Th_2_, Th_9_, T_FH_, Th_17_, or T_reg_ subsets in response to innate immune signals, costimulatory interactions with APCs, paracrine cytokine signals, and through mTOR-mediated changes in energy metabolism [31, 120]. The resulting CD4^+^ T cell subsets are highly plastic with numerous transitory populations identified that are capable of heterogeneous cytokine production as well as the ability to cross talk with other naïve, effector, memory, and regulatory CD4^+^ T cells. With continued stimulation, CD4^+^ T cells develop patterns of stable cytokine expression, yet chromatin remodeling alters cytokine programs in subsets containing bivalent chromatin modifications at loci encoding lineage-specific transcription factors to maintain the capability of shifting their phenotype in response to environmental alterations [32]. mTOR was recently identified as a possible master regulator of CD4^+^ T cell differentiation [26] and exerts CD4^+^ T cell specification through alterations in cellular metabolism. An improved understanding of how to modulate CD4^+^ T cell pools through inducing phenotypic shifts could provide wide health benefits from limiting autoimmune responses to optimizing antitumor immune responses and represents an exciting area of investigation.

## Supplementary Material

Supplemental figure 1: Simplified diagram of CD4+ T cell lineage commitment. Numbers over arrows correspond to types of experiments described in Table 2, which are used to identify the effector molecule following the arrow.
Supplemental table 1: Experiments used to study CD4+ T cell subset specification.

## Figures and Tables

**Table 1:** Characterization of CD4^+^ T cell subsets.

Th subset	Factors inducing lineage	STAT activated	Lineage-specifying transcription factor	Effector cytokines produced	Functions
Th_1_	IL-12 IL-27	STAT4, STAT1	T-bet	IFN*γ*, lymphotoxin, TNF*α*	Cell-mediated immunity, delayed-type hypersensitivity responses, clearance of intracellular pathogens and tumor cells, opsonizing Ab production by B cell class-switching to IgG2a [3, 4, 7–9]

Th_2_	IL-4 Indoleamine 2,3-dioxygenase	STAT6	Gata-3 c-MAF	IL-4, IL-5, IL-13, IL-10	Humoral immunity, clearance of extracellular bacteria and worms, B cell class-switching to IgE, allergic responses [3, 4, 7, 10, 11]

Th_9_	IL-4 TGF*β*	STAT6	BATF	IL-9, IL-10	Protection against parasitic worms/helminth infections [12, 13]

Th_17_	IL-6 MyD88 Low TGF*β* IL-23	STAT3	ROR*γ*T, ROR*α*	IL-17, IL-17F, IL-6, IL-22, TNF*α*, IL-10	Protection of mucosal surfaces, recruitment of neutrophils, clearance of *Mycobacterium tuberculosis* and *Klebsiella pneumonia* [3, 14–17]

Th_22_	IL-10R*β* IL-6 TNF*α*	STAT3	Aryl hydrocarbon receptor	IL-22, IL-13, FGF, CCL15, CCL17, TNF*α*	Mucosal immunity, prevention of microbial translocation across epithelial surfaces, promotes wound repair. [18–21]

Th_25_	IL-4, IL-25	Unknown	Act1	IL-25, IL-4, IL-5, IL-13	Mucosal immunity, stimulates nonlymphoid cells to produce IL-4, limits Th_1_ and Th_17_ induced inflammation, CD4^+^ T cell memory (mouse) [12, 22]

T_FH_	Strong TCR signal, IL-12, CXCR5, IL-21, IL-4	STAT3	MAF (IL-21 transactivator)	IL-21, OX40, ICOS	Helps B cells produce high affinity, class-switched antibodies, guides migration into germinal centers [23, 24]

T_reg_ (includes Tr1 and Th_3_ cells)	High TGF*β* mTOR	STAT5	Foxp3	IL-10, TGF*β*	Suppression of existing immune responses, maintains tolerance/protection against autoimmunity [15, 16, 25, 26]
