# Supplementary material for: Functional and Phenotypic Plasticity of CD4+ T Cell Subsets
Source: Biomed Res Int. 2015 Oct 25;2015:521957. doi: 10.1155/2015/521957 (PMC4637038; doi:10.1155/2015/521957)
Supplement: Supplementary file 1 — Supplemental figure 1: Simplified diagram of CD4+ T cell lineage commitment. Numbers over arrows correspond to types of experiments described in Table 2, which are used to identify the effector molecule following the arrow. Supplemental table 1: Experiments used to study CD4+ T cell subset specification. [file 521957.f1.docx]

Supplemental experimental methods:

The study of CD4^+^ T cell subset specification involves investigation of multiple processes. Early in CD4^+^ T cell lineage commitment, innate immune signals, including cytokines and chemokines, result in activation of signal transducers and activators of transcription (STATs). STAT activation results in activation of lineage specific transcription factors, which results in cytokine production, epigenetic changes at cytokine loci, and subset specification (Supplemental figure 1). Additionally, alteration in metabolic activity of the CD4^+^ T cell contributes to specification and maintenance of each subset, largely influenced by mTOR signaling.

Novel transcription factors can be identified by cloning approaches, including biochemical purification, detection of transcription factors in situ within tissue, and through use of a yeast one-hybrid system. After identification of a transcription factor, it is further characterized through use of DNA binding assays. These include electrophoretic mobility shift assays, DNase I protection assays, methylation interference assays, Southwestern blotting, and through cross-linking with ultraviolet radiation.

Effector cytokines are measured through *in vitro* techniques as well as *in vivo* models. *In vitro* techniques include use of enzyme-linked immunosorbent assays, Elispot assays, flow cytometry for intracellular cytokine measurement, and multiplexed microsphere bead assays for serum cytokine detection. *In vivo* models include *in vivo* cytokine capture assays and use of cytokine reporter mice. In cytokine reporter mice, expression of a cytokine is monitored through expression of a fluorescent-tagged fusion protein.

Interchromosomal interactions are studied by use of chromatin conformation capture techniques and fluorescence *in situ* hybridization. Methylation status is tested by use of methylation-specific PCR identifying specific CpG sites along DNA sequences, bisulphite treatment to identify 5-methylcytosine residues, and use of DNA demethylating agents, including 5-aza-2’-deoxycytidine. When intra- or inter-chromosomal interactions are not identified by the above techniques, genome-wide transcriptional analyses can be utilized. These include chromatin immunoprecipitation, 5-methylcytosine quantitation by high performance liquid chromatography, or through pyrosequencing.

CD4^+^ T cell differentiation is studied both *in vitro* and *in vivo*. *In vitro* techniques include use of fetal thymic organ cultures and cytokine polarization assays. Available *in vivo* models include cytokine transgenic and knockout mice and use of pathogens to induce polarized immune responses.

Several methods can be used to study CD4^+^ T cell subsets, and approaches to identifying transcription factors, analyzing effector cytokine production, measuring epigenetic repression, and studying CD4^+^ T cell differentiation within *in vitro* or *in vivo* environments are described below in supplemental table 1.

Innate immune signals

STAT activation

1.

Activation of lineage-specific transcription factors

2.

Cytokine production

+ feedback

Epigenetic changes at cytokine loci


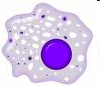


3.

Subset specification

4.

Supplemental figure 1: Simplified diagram of CD4^+^ T cell lineage commitment. Numbers over arrows correspond to types of experiments described in Table 2, which are used to identify the effector molecule following the arrow.

| **1. Identification of novel transcription factors** | | |
| --- | --- | --- |
| *Goal* | *Experimental technique* | *Description and use* |
| Identification – cloning approaches  Reference: [1] | Biochemical purification | Purifies transcription factors from nuclear extracts by ammonium sulfate fractionation and ch**r**omatographic methods |
|  | *In situ* transcription factor detection | Screening of a cDNA expression library using radiolabeled oligonucleotide probes containing transcription factor recognition sites |
|  | Yeast one-hybrid assay | Selection by expression of cDNA clones encoding the transcription factor in yeast as fusion proteins with target activation domains |
| Characterization – DNA binding assays  References: [1-3] | Electrophoretic mobility shift assay | Identifies binding of a sequence-specific DNA binding protein through reduced gel mobility of the DNA-protein complex |
|  | DNase I protection assay | Identifies binding of a protein to a specific region within a DNA fragment |
|  | Methylation interference assay | Identifies binding site of a transcription factor |
|  | Southwestern blotting | Identifies protein-DNA interactions |
|  | UV cross-linking | Linkage of transcription factors to recognition sites on DNA through UV-irradiation |
| **2.** **Measurements of effector cytokine production** | | |
| *In vitro* techniques  References: [4-6] | Enzyme-linked immunosorbent assay (ELISA) | Determines the concentration of cytokine produced by cells in culture through an immunoenzymatic reaction |
|  | Elispot assay | Determines the number of cells secreting a cytokine of interest  *Combined use of ELISA and Elispot is used to calculate the mean production of a cytokine by a single stimulated cell |
|  | Flow cytometry for intracellular cytokine production | Identifies a population of cytokine producing cells by intracellular staining or cytometric bead arrays |
|  | Luminex method for serum cytokines | Uses a flow cytometry-like method with microspheres to quantify sample analytes |
| *In vivo* models  Reference: [7] | *In vivo* cytokine capture assay | Quantifies measurements of cytokines in serum using an ELISA-based method by increasing the cytokine’s *in vivo* half-life |
|  | Cytokine reporter mice | Expression of a cytokine can be monitored through expression of a fusion protein containing a fluorophore. The cytokine locus can be floxed in a CD4^+^ Cre-recombinase system for conditional expression in CD4^+^ T cells |
| **3. Determination of intra- and interchromosomal interactions** | | |
| Interchromosomal interactions  References: [8-10] | Chromatin conformation capture technique | Identifies the juxtaposition frequency between any two genomic loci to colocalize transcribed genes and identify associations between a gene and distal regulatory elements |
|  | Fluorescence *in situ* hybridization | Detects specific DNA sequences on chromosomes or localizes specific mRNAs to define spatial-temporal patterns of gene expression |
| Methylation studies  Reference: [11, 12] | Methylation-specific PCR | Identifies CpG sites on DNA, alternative techniques include use of methylation-sensitive restriction enzymes or southern blotting methylated sequences |
|  | Bisulphite treatment of DNA | Identifies 5-methylcytosine residues, since deamination is slower of methylated sequences |
|  | 5-aza-2’-deoxycytidine treatment | Use of a DNA demethylating agent reactivates silenced genes to study the original molecular pathway |
| Genome-wide transcriptional analyses  References: [11, 13] | Chromatin immunoprecipitation (CHIP) | Determines the mechanism of transcriptional repression by immunoprecipitating chromatin with antibodies |
|  | HPLC or HPCE for 5-methylcytosine | Can be applied for quantification of 5-methylcytosines |
|  | Bisulphite treatment with methylight or pyrosequencing | Detects CpG dinucleotides and aberrant DNA methylation |
| **4. T cell differentiation experiments** | | |
| *In vitro* techniques  References: [14, 15] | Fetal thymic organ culture | A model system to study T cell development and selection *in vitro* in a natural environment where interactions of developing thymocytes with thymic stromal cells are maintained |
|  | Cytokine polarization experiments | Differential cytokines in culture medium can be used to polarize naïve CD4^+^ T cell responses |
| *In vivo* models  References: [15, 16] | Cytokine transgenic or knockout mice | Used to delineate the role of a particular cytokine to CD4^+^ T cell development |
|  | Pathogen polarization experiments | Model organisms can be used to polarize the CD4^+^ T cell repertoire  Th1-priming – Intracellular bacteria or virus  Th2 priming – Extracellular bacteria  Th17 priming – *Mycobacteria*, *Klebsiella*  Th9 priming – Helminth infection |

Supplemental table 1: Experiments used to study CD4^+^ T cell subset specification

References

1. Yang VW. Eukaryotic transcription factors: Identification, characterization and functions. J Nutr. 1998;128(11):2045-51.

2. Garner MM, Revzin A. A gel electrophoresis method for quantifying the binding of proteins to specific DNA regions: Application to components of the escherichia coli lactose operon regulatory system. Nucleic Acids Res. 1981;9(13):3047-60.

3. Galas DJ, Schmitz A. DNase footprinting: A simple method for detection of protein-DNA binding specificity. Nucl.Acids Res. 1978;56:138-44.

4. Czerkinsky CC, Nilsson LA, Nygren H. A solid-phase enzyme-linked immunospot (ELISPOT) assay for enumeration of specific antibody-secreting cells. J Immunol Methods. 1983;65(1-2):109-21.

5. Toedter G, Hayden K, Wagner C, Brodmerkel C. Simultaneous detection of eight analytes in human serum by two commercially available platforms for multiplex cytokine analysis. Clinical and Vaccine Immunology. 2008;15(1):42-8.

6. Morgan E, Varro R, Sepulveda H, Ember JA, Apgar J, Wilson J, et al. Cytometric bead array: A multiplexed assay platform with applications in various areas of biology. Clinical Immunology. 2004;110(3):252-66.

7. Singh RR, Saxena V, Zang S, Li L, Finkelman FD, Witte DP, et al. Differential contribution of IL-4 and STAT6 vs STAT4 to the development of lupus nephritis. Journal of Immunology. 2003;170(9):4818-25.

8. Spilianakis CG, Lalioti MD, Town T, Lee GR, Flavell RA. Interchromosomal associations between alternatively expressed loci. Nature. 2005;435(7042):637-45.

9. Wagner M, Hornt M, Daims H. Fluorescence in situ hybridisation for the identification and characterisation of prokaryotes. Curr Opin Microbiol. 2003;6(3):302-9.

10. Fluorescence in situ hybridization. Nature Methods. 2005;2(3):237-8.

11. Wilson CB, Makar KW, Shnyreva M, Fitzpatrick DR. DNA methylation and the expanding epigenetics of T cell lineage commitment. Semin Immunol. 2005;17(2 SPEC. ISS.):105-19.

12. Zhou L, Chong MMW, Littman DR. Plasticity of CD4+ T cell lineage differentiation. Immunity. 2009;30(5):646-55.

13. Aparicio O, Geisberg JV, Sekinger E, Yang A, Moqtaderi Z, Struhl K. Chromatin immunoprecipitation for determining the association of proteins with specific genomic sequences in vivo. Current protocols in molecular biology / edited by Frederick M.Ausubel ...[et al.]. 2005;Chapter 21.

14. Anderson G, Jenkinson EJ. Use of explant technology in the study of in vitro immune responses. J Immunol Methods. 1998;216(1-2):155-63.

15. Murphy E, Shibuya K, Hosken N, Openshaw P, Maino V, Davis K, et al. Reversibility of T helper 1 and 2 populations is lost after long-term stimulation. J Exp Med. 1996;183(3):901-13.

16. Veldhoen M, Uyttenhove C, van Snick J, Helmby H, Westendorf A, Buer J, et al. Transforming growth factor-β 'reprograms' the differentiation of T helper 2 cells and promotes an interleukin 9-producing subset. Nat Immunol. 2008;9(12):1341-6.
